# Knee meniscal extrusion in a largely non-osteoarthritic cohort: association with greater loss of cartilage volume

**DOI:** 10.1186/ar2132

**Published:** 2007-03-02

**Authors:** Changhai Ding, Johanne Martel-Pelletier, Jean-Pierre Pelletier, François Abram, Jean-Pierre Raynauld, Flavia Cicuttini, Graeme Jones

**Affiliations:** 1 Menzies Research Institute, University of Tasmania, 199 Macquarie Street, Hobart 7000, Australia; 2 Osteoarthritis Research Unit, University of Montreal Hospital Centre, Notre-Dame Hospital, 1560 Sherbrooke St East, Montreal H2L 4M1, Canada; 3 ArthroVision Inc., 1871 rue Sherbrooke Est, Montreal H2K 1B6, Canada; 4 Department of Epidemiology and Preventive Medicine, Monash University Medical School, Commercial Road, Melbourne 3181, Australia

## Abstract

We conducted a longitudinal study (duration 2 years), including 294 individuals (mean age 45 years, 58% female), in order to examine associations between meniscal extrusion, knee structure, radiographic changes and risk factors for osteoarthritis (OA) in a largely non-osteoarthritic cohort. Meniscal extrusion, tibiofemoral cartilage defect score and cartilage volume, and tibial plateau bone area were determined using T1-weighted fat-saturated magnetic resonance imaging. At baseline the presence of medial meniscal extrusion was significantly associated with body mass index (odds ratio [OR] per kg/m^2 ^= 1.13, 95% confidence interval [CI] = 1.02–1.25), past knee injury (positive versus negative history: OR = 3.73, 95% CI = 1.16–11.97), medial tibial bone area (OR per cm^2 ^= 1.37, 95% CI = 1.02–1.85), and osteophytes (OR per grade = 4.89, 95% CI = 1.59–15.02). Two-year longitudinal data revealed that medial meniscal extrusion at baseline was associated with a greater rate of loss of medial tibiofemoral cartilage volume (extrusion versus no extrusion: -1.4%/year; *P *< 0.05) and greater risk for increased medial femoral cartilage defects (OR = 2.59, 95% CI = 1.14–5.86) and lateral tibial cartilage defects (OR = 2.64, 95% CI = 1.03–6.76). However, the latter two associations became nonsignificant after adjustment for tibial bone area and osteophytes. This study suggests that increasing body mass index and bone size, past knee injury, and osteophytes may be causally related to meniscal extrusion. Most importantly, meniscal extrusion at baseline is associated with greater loss of knee cartilage over 2 years, and this seems to be mediated mostly by subchondral bone changes, suggesting extrusion represents one pathway between bone expansion and cartilage loss.

## Introduction

Knee osteoarthritis (OA) is a common chronic disease that is characterized by whole-organ abnormalities [1], including cartilage lesion and loss, osteophytes, synovial and subchondral bone alterations, and meniscal tears and extrusion. Meniscal extrusion is where the meniscus is partially or totally displaced away from or uncovers the tibial articular cartilage [2]. It has been suggested that significant meniscal extrusion, as seen on magnetic resonance imaging (MRI), occurs more often in patients with knee OA than in normal control individuals, either because it causes the OA or because laxity of supporting meniscal structures associated with OA predisposes these patients to meniscal extrusion [2]. Meniscal extrusion has been found to be associated with joint space narrowing [2,3], osteophytosis [4,5], the presence of a chondral lesion [6] and meniscal tear [5,7,8] in cross-sectional studies. Longitudinal studies have confirmed that meniscal extrusion is associated with loss of cartilage volume [7,9] and knee cartilage focal loss [10], as determined by MRI. However, most of these studies were conducted in patients with OA, and there are few reports about the relationship between meniscal extrusion and knee structural change in persons without OA. Furthermore, associations between OA risk factors including age, body mass index (BMI), female sex, knee injury, and genetics and meniscal extrusion are unclear, although a cross-sectional study [2] suggested that meniscal extrusion did not correlate with age and/or weight.

The aim of the present longitudinal study was to describe associations between baseline meniscal extrusion and knee structure, radiographic changes, and OA risk factors in a largely non-OA cohort.

## Materials and methods

### Patients

The study was conducted in Tasmania, Australia, primarily in the capital city of Hobart, from June 2000 until December 2001. Participants were selected from two sources. Approximately half were the adult children of patients who had undergone knee replacement for primary knee OA at any Hobart hospital during the years from 1996 to 2000 (offspring: *n *= 186, age 45 years, 59% female). The parents' diagnosis was confirmed by reference to medical records of the orthopaedic surgeon and the original radiograph where possible. The other participants were control individuals (*n *= 186; age 45 years, 57% female) selected at random from the electoral roll. Those selected were eligible to participate if they had no parent with either a history of symptomatic knee OA or a knee replacement for OA. Individuals from either group were excluded on the basis of contraindication to MRI (including metal sutures, presence of shrapnel, iron filings in the eye and claustrophobia). This study was approved by the Southern Tasmanian Health and Medical Human Research Ethics Committee, and all participants provided informed written consent.

The characteristics of the participants were reported previously [11]. The follow-up study was conducted about 2 years later (mean 2.3 years).

### Anthropometrics

Weight was measured to the nearest 0.1 kg (with shoes, socks and bulky clothing removed) using a set of electronic scales (a single unit; Seca Delta, Model 707; Seca, Hamburg, Germany) calibrated using a known weight at the beginning of each clinic session. Height was measured to the nearest 0.1 cm (with shoes and socks removed) using a stadiometer. Body mass index (BMI; kg/m^2^) was calculated.

### Past knee injury

Our definition of past knee injury was documented in the questionnaire as follows: 'Have you had a previous knee injury requiring non-weight-bearing treatment for more than 24 hours or surgery?'

### Radiography

A standing anteroposterior semiflexed view of the right knee (at 15° flexion) was performed in all participants at baseline and scored individually for osteophytes and joint space narrowing, as described previously [12]. Each of the following four features was scored on a scale from 0 to 3 (0 = normal and 3 = severe): medial joint space narrowing, lateral joint space narrowing, medial osteophytes (femoral and tibial combined) and lateral osteophytes (femoral and tibial combined).

### Magnetic resonance imaging

Magnetic resonance imaging (MRI) of the right knee was performed as described previously [13-15]. Knees were imaged in the sagittal plane on a 1.5 T whole-body magnetic resonance unit (Picker International, Cleveland, OH, USA) using a commercial transmit-receive extremity coil. The following image sequence was used: a T1-weighted fat saturation three-dimensional gradient recall acquisition in the steady state; flip angle 55°; repetition time 58 ms; echo time 12 ms; field of view 16 cm; 60 partitions; 512 × 512 matrix; acquisition time 11 min 56 s; and one acquisition. Sagittal images were obtained at a partition thickness of 1.5 mm and an in-plane resolution of 0.31 × 0.31 mm (512 × 512 pixels). The coronal and axial views were then reformatted.

#### Meniscal extrusion assessment

**Figure 1:**
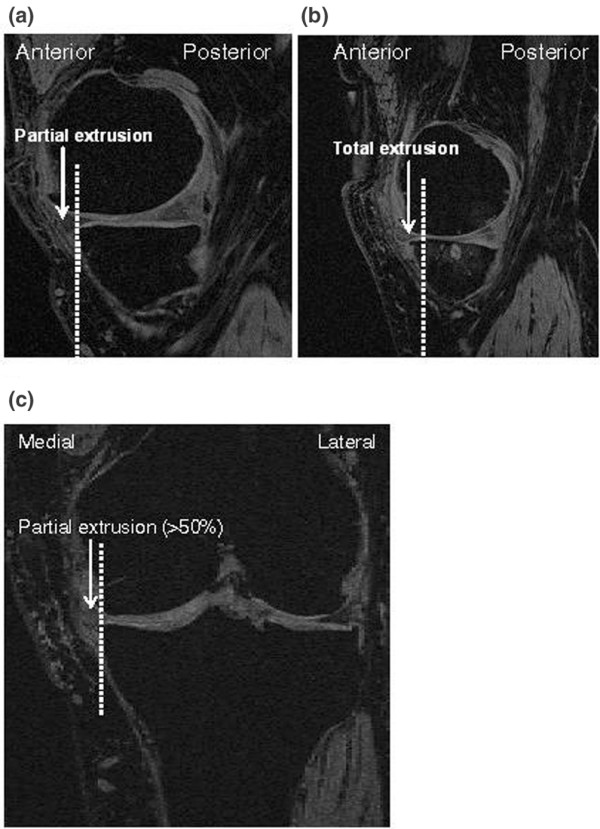
Representations of meniscal extrusion as seen using magnetic resonance imaging. Human knee medial compartment: **(a) **and **(b) **sagittal views, and **(c) **reconstructed coronal view. Partial and total meniscal extrusion at the anterior horn are shown in panels a and b, respectively. Panel c shows a partial (> 50%) meniscal extrusion at the body site.

The extent of meniscal extrusion (Figure 1) on the medial or lateral edges of the tibial femoral joint space, not including the osteophytes, was evaluated at baseline and at 2 years by two observers for the anterior, body, and posterior horns of the menisci. A score from 0 to 2 was employed, in which 0 = no extrusion, 1 = partial meniscal extrusion, and 2 = complete meniscal extrusion with no contact with the joint space. The intra-reader and inter-reader correlation coefficient ranged from 0.85 to 0.92 for the meniscal extrusion, as reported previously [7,9].

#### Knee cartilage volume, defects and bone area measurement

Knee cartilage volume, defects and bone area were evaluated at baseline and 2 years by one observer (CD) who was blinded to the meniscal assessment. Knee cartilage volume was determined by means of image processing on an independent workstation at baseline and follow up. The volumes of individual cartilage plates (medial tibia and femora, and lateral tibia and femora) were isolated from the total volume by manually drawing dis-articulation contours around the cartilage boundaries on a section by section basis. These data were then resampled by means of bilinear and cubic interpolation (area of 312 × 312 μm by 1.5 mm thickness, continuous sections) for the final three-dimensional rendering. The coefficients of variation were 2.1% to 2.2% [13] for tibial cartilage volume measures and were 1.2% to 2.6% [16] for femoral cartilage measures. The percentage change in cartilage volume per year was calculated as follows: (100 × [(cartilage volume at follow up – cartilage volume at baseline)/cartilage volume at baseline]/time between scans in years).

The cartilage defects (scale from 0 to 4) were graded by two observers at medial tibial and femoral, and lateral tibial and femoral sites, as previously described, with excellent reproducibility [17]: grade 0 = normal cartilage; grade 1 = focal blistering and intracartilaginous low-signal intensity area with an intact surface and bottom; grade 2 = irregularities on the surface or bottom and loss of thickness of less than 50%; grade 3 = deep ulceration with loss of thickness of more than 50%; and grade 4 = full-thickness chondral wear with exposure of subchondral bone. Intraobserver reliability (expressed as intraclass correlation coefficient) was 0.89 to 0.94 and interobserver reliability was 0.85 to 0.93 [15,17]. Changes in tibial and/or femoral cartilage defects were calculated by subtracting tibial and/or femoral cartilage defect scores (0 to 4 or 0 to 8 scale) at baseline from tibial and/or femoral cartilage defect scores (0 to 4 or 0 to 8 scale) at follow up.

The area of medial and lateral tibial plateau bone was measured manually on the three reformatted images closest to tibial cartilage. An average of these three areas was used as an estimate of the tibial plateau bone area [12,15]. The coefficients of variation for these measures range from 2.2% to 2.6% [13].

### Data analysis

Unpaired *t*-test or χ^2 ^test was utilized for comparison of means or frequencies. Multiple logistic regression analysis was used to examine the associations between the presence or absence of medial meniscal extrusion at baseline and age, sex, family history of OA, past knee injury, BMI, tibial cartilage volume, cartilage defect scores, tibial bone area, radiographic assessment, and change in cartilage defect score before and after adjustment for confounders. Linear regression was utilized to examine the associations between change in cartilage volume and presence of medial meniscal extrusion at baseline before and after adjustment for confounders. A *P *value less than 0.05 (two-tailed) was considered statistically significant. All statistical analyses were performed on SPSS version 12.0 for Windows (SPSS Inc., Chicago, IL, USA).

## Results

A total of 325 individuals (58% female) completed the study, with a response rate of 87%. The reasons for loss to follow up (and number of individuals) were as follows: deceased (two); moved interstate (five); claustrophobic (three); and illness (four) and others (no reason). Meniscal extrusion was measured in the first 294 participants (58% female; offspring *n *= 135, control individuals *n *= 159) at both baseline (79% of all participants) and follow up (90% of all participants). There were no significant differences in demographic or structural factors between the whole cohort and the individuals who were not included (data not shown).

Meniscal extrusion was found at baseline in 23 participants, with 21 (7% of the total population) at the medial compartment (17 partial at the body site, one partial at body and anterior horn site, and three complete at body site) and two (0.7%) at the lateral compartment (one partial body site and one complete body site). Medial meniscal extrusion was present in 4% of individuals without any radiographic changes (*n *= 242) but in 21% of those with radiographic changes (*n *= 52; *P *< 0.001). There was no difference in presence of medical meniscal extrusion between control individuals without a family history of OA (6%) and offspring with a family history of OA (9%; *P *= 0.28).

Over 2 years, meniscal extrusion developed in only four participants (1.4%; all at the body site in the medial compartment); their mean age was 47 years (range 41 to 54 years) and mean BMI was 32 kg/m^2 ^(range 26 to 45 kg/m^2^). Three of these four individuals were women; three were offspring of patients with severe knee OA, and one had a past knee injury. Because of the small sample size (*n *= 4), the precise reasons for the occurrence of a new meniscal extrusion could not be determined.

The characteristics of the participants are presented in Table 1. The data show that the participants with and those without medial meniscal extrusion were similar in terms of age, sex, family history of OA, height, prevalence of knee pain, baseline cartilage volume, and change in tibial bone area. However, compared with participants with no medial meniscal extrusion, the following parameters were greater in those with meniscal extrusion: BMI, weight, proportion of past knee injury, tibial bone area at baseline, medial tibiofemoral cartilage defect score, prevalence of radiographic OA, medial joint space narrowing and osteophytes at baseline, loss of medial tibial cartilage volume, and progression of tibiofemoral cartilage defect score.

**Table 1:** Characteristics of subjects with and without baseline medial meniscal extrusion

Characteristic	Meniscal extrusion at baseline		*P *value
		
	Negative (*n *= 273)	Positive (*n *= 21)	
Age (years)	45.0 (6.6)	47.2 (5.4)	0.148
Sex (female [%])	59	43	0.150*
Family history of OA (positive [%])	45	57	0.284*
Height (cm)	169.0 (8.5)	172.1 (8.2)	0.102
Weight (kg)	76.6 (14.6)	92.7 (21.5)	< 0.001
BMI (kg/m^2^)	26.8 (4.5)	31.3 (7.0)	< 0.001
Knee pain (%)	33	38	0.665*
Obese (%)	18	52	< 0.001*
Past knee injury (%)	19	38	0.037*
Medial tibial cartilage volume (ml)	2.2 (0.5)	2.4 (0.5)	0.051
Lateral tibial cartilage volume (ml)	2.6 (0.7)	2.7 (0.6)	0.408
Medial femoral cartilage volume (ml)	4.6 (1.3)	4.7 (1.0)	0.632
Lateral femoral cartilage volume (ml)	4.7 (1.3)	5.1 (1.0)	0.280
Medial tibial bone area (cm^2^)	17.3 (2.6)	19.8 (3.8)	< 0.001
Lateral tibial bone area (cm^2^)	11.9 (2.0)	13.5 (2.5)	0.001
Medial tibiofemoral cartilage defect (0–8)	2.0 (0.6)	3.1 (1.4)	< 0.001
Lateral tibiofemoral cartilage defect (0–8)	2.0 (0.7)	2.1 (0.7)	0.550
Any radiographic osteoarthritis (%)	15	52	< 0.001*
Medial joint space narrowing (%)	13	38	0.001*
Medial osteophytes (%)	4	43	< 0.001*
Lateral joint space narrowing (%)	3	5	0.737*
Lateral osteophytes (%)	3	14	< 0.001*
Change in medial tibial cartilage volume (%)	-2.4 (4.1)	-4.7 (4.4)	0.015
Change in lateral tibial cartilage volume (%)	-1.5 (3.4)	-1.6 (3.8)	0.860
Change in medial femoral cartilage volume (%)	-3.3 (2.6)	-4.1 (3.9)	0.278
Change in lateral femoral cartilage volume (%)	-3.3 (2.5)	-3.5 (3.1)	0.662
Change in medial tibial bone area (%)	+0.7 (1.8)	+0.2 (1.7)	0.258
Change in lateral tibial bone area (%)	+0.3 (2.8)	+0.5 (2.4)	0.781
Change in medial tibiofemoral cartilage defect	-0.07 (0.86)	0.29 (1.06)	0.074
Change in lateral tibiofemoral cartilage defect	-0.02 (0.81)	0.50 (1.20)	0.007

Values are expressed as mean (standard deviation), except for percentage. *χ^2 ^test; all others *t*-test. BMI, body mass index; OA, osteoarthritis.

Age, sex and family history of OA were not significantly associated with baseline medial meniscal extrusion in both univariable and multivariable analyses (Table 2). However, BMI, weight, obesity and our definition of past knee injury were significantly associated with baseline medial meniscal extrusion, even after adjustment for confounders (Table 2). Specifically, medial meniscal extrusion occurred in 18% of obese participants (BMI = 30 kg/m^2^; *n *= 61) versus 4% of nonobese participants (BMI < 30 kg/m^2^; *n *= 233), and 13% of those with past knee injury (*n *= 60) versus 6% of those without past knee injury (*n *= 234). Moreover, baseline medial meniscal extrusion was significantly associated with medial tibial bone area and medial osteophytes, but was not associated with medial tibial cartilage volume both in univariable and multivariable analyses. It was associated with medial joint space narrowing (19% versus 5% medial meniscal extrusion in participants with and without medial joint space narrowing) before (Table 2) and after adjustment for age, sex, family history of OA, BMI, our definition of past knee injury and baseline medial tibial cartilage volume (odds ratio = 3.86; *P *= 0.021), but this association became nonsignificant after further adjustment for tibial bone area and osteophytes (Table 2).

**Table 2:** Factors associated with baseline medial meniscus extrusion

Factor	Univariable (OR [95% CI])	Multivariable^a ^(OR [95% CI])	*P *value
Age (per year)	1.06 (0.98–1.13)	1.03 (0.94–1.13)	0.579
Sex (female versus male)	0.52 (0.21–1.28)	3.67 (0.65–20.80)	0.141
OA family history (+ versus -)	1.63 (0.66–3.99)	0.65 (0.21–2.02)	0.452
BMI (per unit)	1.16 (1.07–1.25)*	1.13(1.02–1.25)*	0.019
Weight (per kg)	1.06 (1.03–1.09)*	1.04 (1.00–1.08)*^b^	0.029
Obese (+ versus -)	4.90 (1.98–12.18)*	4.87 (1.59–14.92)*^c^	0.006
Past knee injury (+ versus -)	2.62 (1.03–6.64)*	3.73 (1.16–11.97)*	0.027
Medial tibial cartilage volume (per ml)	2.06 (0.99–4.31)	1.52 (0.42–5.54)	0.527
Medial tibial bone area (per cm^2^)	1.35 (1.15–1.59)*	1.37 (1.02–1.85)*	0.038
Medial radiographic osteoarthritis (+ versus -)	6.88 (2.57–18.40)*	4.21 (1.25–14.21)*	0.021
Medial joint space narrowing (per grade)	4.27 (1.65–11.06)*	1.97 (0.56–6.87)	0.288
Medial osteophytes (per grade)	9.52 (3.49–25.97)*	4.89 (1.59–15.02)*	0.006

The dependent variable was medial meniscal extrusion. ^a^Adjusted by factors listed in the table. ^b^Adjusted for height and factors listed except for body mass index (BMI). ^c^Adjusted for factors listed except for BMI and weight. *Statistically significant associations. CI, confidence interval; OA, osteoarthritis; OR, odds ratio.

Data from Table 3 show that baseline medial meniscal extrusion was significantly associated with baseline medial tibial, femoral and tibiofemoral cartilage defect scores after adjustment for age, sex, family history of OA, BMI, past knee injury and change in cartilage defect scores, and with change in medial femoral and lateral tibial cartilage defect scores after adjustment for age, sex, OA family history, BMI, past knee injury, and baseline cartilage defect scores (Table 3). However, these associations again became nonsignificant after further adjustment for baseline tibial bone area and osteophytes (Table 3).

**Table 3:** Associations between baseline medial meniscal extrusion and baseline cartilage defect score and change in cartilage defect score over 2 years

Factor	Multivariable^a ^(OR [95% CI])	Multivariable^b ^(OR [95% CI])	Multivariable^c ^(OR [95% CI])
Baseline cartilage defect score			
Medial tibial (0–4)	5.91 (2.53–13.77)*	3.41 (1.36–8.57)*	0.63 (0.17–2.32)
Medial femoral (0–4)	6.71 (2.65–16.99)*	4.62 (1.74–12.30)*	2.39 (0.71–8.02)
Medial tibiofemoral (0–8)	3.42 (1.92–6.10)*	2.45 (1.36–4.40)*	1.23 (0.59–2.53)
Lateral tibial (0–4)	1.52 (0.59–3.98)	0.97 (0.29–3.22)	0.20 (0.03–1.37)
Lateral femoral (0–4)	1.84 (0.78–4.34)	1.30 (0.45–3.74)	0.49 (0.12–2.05)
Lateral tibiofemoral (0–8)	1.33 (0.82–2.17)	1.13 (0.63–2.05)	0.55 (0.22–1.39)
Change in cartilage defect score			
Medial tibial (0–4)	1.54 (0.57–4.13)	1.24 (0.45–3.44)	0.31 (0.07–1.42)
Medial femoral (0–4)	3.12 (1.50–6.49)*	2.59 (1.14–5.86)*	1.30 (0.51–3.35)
Medial tibiofemoral (0–8)	1.79 (1.06–3.03)*	1.56 (0.88–2.77)	0.85 (0.40–1.81)
Lateral tibial (0–4)	2.92 (1.25–6.79)*	2.64 (1.03–6.76)*	1.54 (0.48–4.95)
Lateral femoral (0–4)	2.30 (1.10–4.81)*	1.68 (0.74–3.79)	1.01 (0.39–2.67)
Lateral tibiofemoral (0–8)	2.01 (1.22–3.31)*	1.80 (1.05–3.08)*	1.21 (0.61–2.42)

The dependent variable was medial meniscal extrusion. ^a^Adjusted for change in cartilage defect score if associations between baseline cartilage defect scores and baseline meniscal extrusion were determined, or baseline cartilage defect score if associations between changes in cartilage defect score and baseline meniscal extrusion were determined. ^b^Further adjusted for age, sex, offspring/control status, body mass index and past knee injury. ^c^Further adjusted for baseline tibial bone area and osteophytes. *Statistically significant associations. CI, confidence interval; OR, odds ratio.

Prevalent medial meniscal extrusion at baseline was significantly associated with subsequent rate of change in medial femoral and tibiofemoral cartilage volume after adjustment for the confounders (Table 4). In contrast, medial meniscal extrusion at baseline was significantly associated with change in medial tibial cartilage volume in univariable analysis (Table 4), but this association became nonsignificant after adjustment for other factors, mostly contributed by BMI and baseline cartilage volume (β = -1.41%; *P *= 0.128).

**Table 4:** Associations between medial meniscal extrusion at baseline and change in cartilage volume over 2 years

Factor	Univariable β (95% CI)	Multivariable^a ^β (95% CI)	Multivariable^b ^β (95% CI)
Medial tibial (%)	-2.29 (-0.41 to -0.45)*	-1.26 (-2.95 to +0.43)	-1.01 (-2.88 to +0.86)
Medial femoral (%)	-0.73 (-2.06 to +0.59)	-0.87 (-2.23 to +0.49)	-1.56 (-2.99 to -0.14)*
Medial tibiofemoral (%)	-1.42 (-2.66 to -0.17)*	-1.18 (-2.41 to +0.05)	-1.44 (-2.76 to -0.12)*
Lateral tibial (%)	-0.14 (-1.69 to +1.41)	+0.43 (-1.09 to +1.94)	+0.64 (-0.94 to +2.23)
Lateral femoral (%)	-0.28 (-1.54 to +0.98)	-0.34 (-1.62 to +0.94)	-0.27 (-1.61 to +1.07)
Lateral tibiofemoral (%)	-0.28 (-1.45 to +0.88)	-0.07 (-1.22 to +1.09)	+0.15 (-1.05 to +1.35)

The dependent variable was change in cartilage volume. ^a^Adjusted for age, sex, offspring-control status, body mass index, past knee injury and baseline cartilage volume. ^b^Further adjusted for baseline tibial bone area and osteophytes. *Statistically significant associations. CI, confidence interval.

## Discussion

To our knowledge, this is the first time that associations of meniscal extrusion with knee structural changes and risk factors in a largely non-OA cohort have been documented. As may be expected for such individuals, the prevalence of meniscal extrusion was low, predominantly involving partial extrusion. Medial meniscal extrusion was significantly associated with BMI, past knee injury and tibial bone area, as well as with osteophytes and medial tibiofemoral cartilage defect scores. Over 2 years, those with medial meniscal extrusion exhibited greater loss of medial tibiofemoral cartilage volume and greater increase in medial femoral and lateral tibial cartilage defect score than did those without medial meniscal extrusion. The association between meniscal extrusion and knee cartilage defects became nonsignificant after adjustment for bone area and/or osteophytes, suggesting that extrusion may represent one pathway between bone expansion and cartilage loss.

In this largely non-OA cohort, medial and lateral meniscal extrusion occurred in 7% and 0.7% of individuals, respectively, and medial meniscal extrusion developed in 1.4% over 2 years. These prevalence rates are similar to those observed in normal individuals [4] and those with knee pain [18], but far less than those observed in patients with either symptomatic or asymptomatic knee OA [2] and in nonadvanced arthritic patients who underwent arthroscopy for other disorders [6]. The difference is most likely due to different disease status and age group in the different cohorts. In the present study, individuals with early radiographic changes had a greater prevalence of medial meniscal extrusion than did those with no radiographic changes.

Any factors that affect meniscal stability and structure may cause meniscal extrusion. In this longitudinal study only four participants developed partial meniscal extrusion over 2 years, so we could not document any predictors of meniscal extrusion. However, cross-sectional data from this study show that although age and family history of OA were not associated with medial meniscal extrusion, our definition of past knee injury and weight (body weight, BMI and obesity) were significantly associated with prevalent medial meniscal extrusion. Obese individuals had nearly a fivefold increased risk for having meniscal extrusion than did nonobese individuals, and those satisfying our definition of past knee injury had a nearly fourfold increase in risk for having meniscal extrusion compared with those with no such history. These suggest that obesity and past knee injury are major risk factors for meniscal extrusion. It is possible that knee injury was in part caused by chronic knee instability resulting from an overuse of secondary stabilizers such as cruciate and collateral ligaments [19]. Moreover, women tended to be at greater risk for having medial meniscal extrusion than did men in multivariable analysis, although this did not reach statistical significance.

We have reported that tibial bone area and osteophytes are strongly associated with prevalent knee cartilage defects [17] and incident knee cartilage defects [15]. We found in this study that medial tibial bone area and osteophytes were also significantly associated with the prevalence of medial meniscal extrusion, suggesting that subchondral bone expansion may play an important role in the initiation not only of cartilage damage [15,17] but also of meniscal extrusion. It is also possible that meniscal extrusion can induce subchondral bone expansion and osteophytes, because without the meniscus, which functions as an energy absorber, the increased contact between tibia and femur may contribute to remodelling of bone. However, we found that baseline medial meniscal extrusion was not associated with change in tibial bone area (Table 1), suggesting that tibial bone area is not affected during the early stages of meniscal extrusion.

With increasing meniscal extrusion, there may be increasing contact stress on the tibial and femoral articular cartilage, which theoretically might accelerate the development of cartilage damage. Berthiaume and coworkers [7] reported that OA patients with severe medial meniscal tear had greater loss of medial compartment cartilage volume than did those with no medial meniscal tear over 2 years. Raynauld and coworkers [9] further reported that severe meniscal extrusion was more prevalent (73%) in OA patients who experienced more rapid loss of global knee cartilage volume, and severe medial meniscal extrusion was found to be an independent predictor of loss of medial compartment cartilage volume over 2 years. Hunter and coworkers [10] reported that meniscal malposition was significantly associated with increased risk for focal cartilage loss over 30 months in patients with symptomatic OA. Consistent with these findings in patients with disease, in the present study – conducted largely in individuals without OA – we found medial meniscal extrusion to be significantly associated with change in medial femoral and lateral tibial cartilage defects over 2 years after adjustment for age, sex, BMI, family history of OA and past knee injury. Furthermore, we found that medial meniscal extrusion was significantly associated with loss of medial tibiofemoral cartilage volume over 2 years after adjustment for the above factors as well as bone changes.

Our data suggest that tibial bone area and osteophytes may lead to meniscal extrusion, which in turn leads to joint space narrowing on radiography and knee cartilage defects and tibial cartilage loss. We found that meniscal extrusion was significantly associated with joint space narrowing in the unadjusted analysis and after adjustment for age, sex, OA family history and BMI. This is consistent with previous findings showing that meniscal extrusion (rather than meniscal compression) contributes to joint space narrowing [2,3,9]. However, the association between meniscal extrusion and joint space narrowing became nonsignificant after adjustment for tibial bone area and osteophytes. This suggests that subchondral bone changes leads to meniscal extrusion and joint space narrowing. Consistent with this, after adjustment for tibial bone area and osteophytes, the associations between meniscal extrusion and prevalent and incident defects in knee cartilage disappeared, and the association between meniscal extrusion and tibial cartilage loss decreased in magnitude. This suggests that meniscal extrusion is an intermediate variable on the pathway between bone change and cartilage damage.

The study has a number of potential limitations. It was primarily designed to examine genetic mechanisms of knee OA and utilized a matched design. The matching protocol was not adhered to in the present study, but adjustment for family history did not alter the results. Although the sample is a convenience sample, Miettinen [20] stated that for associations to be generalized to other populations, three key criteria including selection (inclusion/exclusion criteria for both offspring and controls are explicitly defined), sample size and adequate distribution of study factors need to be met, all of which are met by this study. Second, we cannot confirm the cross-sectional results in the longitudinal component of our study because of the low incidence rate; hence, studies of longer term will be required. Third, the amount of meniscal extrusion may be underestimated on MRI scans because of non-weight bearing knees and may have been more prevalent if the study had been carried out under load conditions. Pathological patterns such as joint laxity, axial deviation or varus-valgus deformity may also influence the measured values under non-weight-bearing conditions. Fourth, we asked all participants to wait for 15 min in the waiting room before MRI scan, but we did not record the intensity of load force and recovery time after running, walking, or sitting. This may influence the measurement of cartilage volume. However, a recent study [21] showed that these moderate activities had little impact on tibial and femoral cartilage deformation *in vivo *in healthy individuals. A fifth limitation is that our definition of past knee injury can be considered imprecise. However, we found in this study that it was significantly associated with meniscal extrusion, suggesting that the definition has predictive validity. Finally, measurement error may influence the results. However, scoring of meniscal extrusion, knee cartilage defects, volume, bone size and tibiofemoral radiographic score measurement was highly reproducible, suggesting this is unlikely.

## Conclusion

This study suggests that increasing BMI and bone size, past knee injury, and osteophytes may be causally related to meniscal extrusion. Most importantly, meniscal extrusion at baseline is associated with greater loss of knee cartilage over 2 years and this association is mostly mediated by subchondral bone changes, suggesting that extrusion represents one pathway between bone expansion and cartilage loss.

## Abbreviations

BMI = body mass index; MRI = magnetic resonance imaging; OA = osteoarthritis.

## Competing interests

The authors declare that they have no competing interests.

## Authors' contributions

CD, GJ, JM-P, J-PP, J-PR and FC participated in the design of the study. CD and FA carried out the measurement of cartilage volume, cartilage defects, bone size and/or meniscal extrusion. CD performed the statistical analysis and drafted the manuscript. All authors reviewed the manuscript, and read and approved the final manuscript.
